# Compliance of medical practitioners with diabetic treatment guidelines in West Rand, Gauteng

**DOI:** 10.4102/safp.v65i1.5633

**Published:** 2023-03-23

**Authors:** Nneka J. Ohanson, Deidré Pretorius

**Affiliations:** 1 Division of Family Medicine, School of Clinical Medicine, University of the Witwatersrand, Johannesburg, South Africa

**Keywords:** compliance, diabetes, medical practitioners, treatment guidelines, Society for Endocrinology Metabolism and Diabetes South Africa (SEMDSA) guidelines

## Abstract

**Background:**

Diabetes mellitus is increasing globally and is associated with multiple complications. Guidelines have been formulated to standardise care among people living with diabetes mellitus (DM), but research shows poor compliance with treatment guidelines. The aim of this study was to assess how well healthcare practitioners in a district hospital in Gauteng complied with the most recent diabetic treatment guideline, Society for Endocrinology Metabolism and Diabetes South Africa (SEMDSA) 2017.

**Methods:**

A retrospective cross-sectional review of patient record living with diabetes was done. This study was conducted in the out-patient department of Dr Yusuf Dadoo hospital in the West Rand, Gauteng. A total of 323 records of patients seen from August 2019 to December 2019 were reviewed, and some of the basic variables were assessed according to the most recent diabetic treatment guidelines SEMDSA 2017.

**Results:**

Files were audited in four categories: comorbidities, examinations, investigations and the presence of complications. Six monthly glycated haemoglobin (HbA1c) was assessed in 40 (12.4%), annual creatinine assessed in 179 (55.4%) and lipogram in 154 (47.7%) of patients. More than 70% of patients had uncontrolled glycaemia and two people were screened for erectile dysfunction.

**Conclusion:**

Monitoring and control parameters were infrequently done as per guideline recommendations. The resultant effects were poor glycaemic control and thus numerous complications.

**Contribution:**

Targeted strategies to improve medical practitioner compliance to guidelines including further research to study factors associated with poor compliance with guidelines are needed to improve the overall care of people living with DM in the West Rand and thus minimise the risk of complications among patients in the district.

## Introduction

There has been a rise in the prevalence of diabetes mellitus (DM) in Africa and sub-Saharan Africa (SSA) due to increasing urbanisation and economic development.^1^ About 415 million people are living with DM globally, and the estimated national prevalence in South Africa is estimated at 15.25%.^2^

The increase in the prevalence could probably be due to the increasing urbanisation and economic development in the region.^3^ The increase in rural-urban migration has led to changes in food and diet. The difference in dietary trends has moved from fresh foods to over-processed and canned food.^4^ In addition to change in dietary trends, the corresponding increases in a sedentary lifestyle predispose to obesity a significant risk factor for diabetes.^4^ The current human immunodeficiency virus (HIV) epidemic in SSA and with associated increasing use of antiretroviral therapy also increase the risk of insulin resistance.^5^ Furthermore, the effects of the coronavirus disease 2019 (COVID-19) pandemic with its seemingly bidirectional relationship with diabetes increase the number of patients living with diabetes in South Africa.^6^ In addition, the presence of DM significantly increases the risk of severe disease and mortality compared with people without DM.^7^

A systematic review of 49 articles assessing the quality of primary care on diabetic outcomes has found that high-quality primary health care, associated with clinical guideline compliance, significantly reduced hospital admissions and complications for people living with DM.^8^ Quality clinical care is important, and therefore the question was raised to what extent did doctors comply with guidelines in the management of patients living with diabetes in West Rand Health District, Gauteng.

Clinical guidelines are systematically developed recommendations that assist medical practitioners in making informed clinical decisions to improve the management of diabetes. They are derived by synthesising high-quality scientific evidence regarding specific aspects of patient care.^9^ Benefits and risks are weighed against the evidence gathered and recommendations are translated into guidelines. The objectives of clinical care guidelines, therefore, are to standardise and ensure uniformity of patient care, thereby improving the quality of patient care and minimising risks.^9,10^ Healthcare compliance therefore refers to the process of abiding with all legal, professional and ethical standards in healthcare,^11^ and adherence is the act, action or quality of adhering to this process.

In South Africa, the Society for Endocrinology Metabolism and Diabetes South Africa (SEMDSA) has formulated clinical guidelines to provide guidance on the most appropriate management for people with diabetes. In addition, to enhance diabetes prevention efforts, reduce the burden and complications of the disease and inform clinical decisions made by healthcare practitioners.^12^

Clinical guidelines are readily available in various clinical settings in South Africa. However, despite the advances in scientific evidence-based clinical recommendations, several studies show that physicians complied poorly with diabetic treatment guidelines.^13,14^ Treatment gaps have been found in various other studies regarding the management of DM among patients locally and abroad, and they have also been found to be associated with poor compliance with clinical guidelines.^15,16^

In Norway and Switzerland, the researchers discovered significant discrepancies between the laid-down clinical guidelines and the practices of healthcare practitioners.^17,18^ In addition, the Diab–Africa project, done across six countries in sub-Saharan Africa, has found that less than half of patients had glycaemic levels and monitoring parameters assessed in the study year.^19^ Similarly, Kibirige et al^20^ in Uganda has found that glycaemic, blood pressure control and screening for diabetic complications were poorly done by healthcare workers.

In two South African studies, it was found that healthcare practitioners complied poorly with the recommended diabetes treatment guidelines. In a district hospital in KwaZulu-Natal, poor health outcomes were mainly contributed to poor compliance with the current diabetic guidelines.^21^ In Kwa-Zulu-Natal, only 25% of patients had their HbA1c levels done the preceding year, and in Tshwane, Pretoria, there was infrequent monitoring of glycaemia recorded in the audited files (half of which had no blood glucose recorded, and more than 80% had no urine testing recorded).^21,22^ The National Development Plan aimed already in 2017 to reduce the disease burden to manageable levels and decrease medico-legal risks and litigation.^23,24^ Guidelines protect doctors and following them curb costs by decreasing the burden of disease and its complications. They prevent additional diagnostic examinations but also decrease the risk of litigation.^23,24^

The researcher observed clinical inconsistencies and sub-optimal patient care in her workplace that were anecdotally attributed to possible poor compliance to clinical guidelines, burnout and excessive workloads. The concern is that a lack of standardised care would lead to numerous complications among these patients which by itself is not only to the detriment of the patient but also increases the work burden. The need to assess compliance to guidelines was identified. In addition, this seems to be the first study assessing compliance with the guidelines in this district. The aim of this study, therefore, was to assess how well medical practitioners working at a district hospital in West Rand complied with the most recent diabetic treatment guidelines (SEMDSA 2017).^12^

## Methods

### Study design

A retrospective cross-sectional review of patient records was done.

### Setting

The setting was the Out-patient Department (OPD) of Dr Yusuf Dadoo Hospital, Krugersdorp, in the Mogale Municipality in the West Rand Health District of Gauteng with a population of about 383 864 (according to the 2016 population survey). An average of 10–15 patients with diabetes are seen daily at this OPD.

### Sampling

The target population were adult patients living with diabetes attending regular follow-up at the OPD at this hospital. The files of patients living with diabetes older than 18 years at this OPD clinic between April 2018 and March 2019 were estimated to be about 2112 per annum by the District Health Information System information. The sample was calculated with Epi-info software version 7.^25^ Using a confidence interval of 95% and a sample error of 5%, 343 files were assessed while 20 files were discarded based on the exclusion criteria.

The records of patients seen from 01 August 2019 to 31 December 2019 were eligible for review. Convenience sampling – a non-probability sampling method – was used where every file on the Diabetes OPD register was audited in a consecutive manner until the sample size was reached. As patients consulted more than once during the sampling period, repeat files were only captured as one file, and the most relevant version during the selected period was audited. Some of the most basic variables were assessed according to the most recent diabetic treatment guidelines SEMDSA 2017. Selection was done using the diabetes daily register of patients in the OPD. Medical records of patients that presented for ambulatory care and who were diagnosed for at least 1 year with diabetes were included in the study. Files of patients seen during weekends and pregnant diabetics were excluded from the study. Files not meeting inclusion criteria were discarded, and the next file was selected until sample size was reached. Where a file could not be traced, the next file was selected.

### Data collection

The OPD register was used as a primary source of patient records, whereafter the administrative staff were asked to retrieve files. The researcher then assessed whether the files met the inclusion criteria, and a tracking list was completed. When a file met the inclusion criteria, the researcher included this in the data collection list; thereafter, the file was marked as being audited and returned to the archives. The 2017 SEMDSA^12^ guideline was used to develop the tool and variables were extracted from the document. The patient profile of reviewed documents included the following variables selected from the guideline:

- Comorbid conditions
- Presence of complications
- Routine examination: for feet, blood pressure, waist circumference, body mass index (BMI), weight
- Medications
- Routine investigations to screen for presence of target organ damage: for example, glucose testing at each visit, 3-6 monthly glycated haemoglobin (HbA1c), annual lipogram, annual serum creatinine and estimated glomerular filtration rate (eGFR), urine dipsticks, urine albumin creatinine ratio and echocardiogram.

### Data analysis

Data were captured onto an Excel spreadsheet and analysed using STATA version 16 (StataCorp, College Station, Texas, United States). Descriptive analyses were used to summarise the data, and results were presented in tables.

### Ethical considerations

Permission to conduct the study was obtained from the Human Medical Ethics Committee University of the Witwatersrand (M200117) and the Gauteng Health Research Committee Database (GP_202007_010). No identifiers of persons were used; thus, all data were anonymous and confidential. Data will be stored safely for a period of 2 years after publication of the results, after which it will be discarded in a safe manner.

## Results

A total of 343 files were reviewed, while the 20 files that did not meet the inclusion criteria were discarded. Therefore, 323 patient files were reviewed. This translates to the records of 165 (51%) women and 158 (48.9%) men who were reviewed (Table 1). The mean age of the patients was 60 years, and more than half (195; 60.4%) were unemployed.

**TABLE 1:** Patients profile as documented in the reviewed files (*N* = 323).

Demographics	Categories	Frequency	Percentage
Gender	Women	165	51.1
	Men	158	48.9
Employment	Employed	128	39.6
	Unemployed	195	60.3
Hypertension	Yes	280	86.7
Current medication	Oral agents	187	57.9
	Insulin only	35	10.8
	Oral and Insulin	144	44.6
	Simvastatin	180	55.7

In terms of comorbidity, hypertension was the most frequently documented condition (Table 1). A total of 280 (86.7%) patients lived with hypertension, 27 (8.4%) had arthritis, 23 (7.1%) had gout and 21 (31.7%) had asthma and chronic obstructive disease as comorbidities. In addition, 180/323 (55.7%) were taking oral cholesterol-lowering therapy. This is shown in Table 1.

Furthermore, it should also be noted that only 36/323 (11%) patients were referred to annual eye assessment to either optometry or ophthalmology, but the results of these assessments were not documented in patient records. Only 28/323 (8.7%) documented foot examinations were done. In addition, the SEMDSA guideline requires referral to a dietician and adequate health education by the attending physician; however, only 129 (39.9%) patients were referred to a dietician and 204 (63.4%) had documented health education.

In terms of the documented routine examinations, the analyses of all the files reviewed (100%) had blood pressure measurements done and recorded. However, other examinations – foot exam, fundoscopy, weight, BMI and injection sites – were markedly absent (see Table 2).

**TABLE 2:** Documented routine examinations in reviewed files (*N* = 323).

Examination	Frequency	Percentage
Blood pressure	322	99.7
BMI	38	11.8
Weight	37	11.5
Fundoscopy	36	11.1
Waist circumference	8	2.5
Injection sites	7	2.2

BMI, body mass index.

In addition, the most frequently documented complication was diabetic foot complications that included neuropathy, foot-ulcers and gangrene for 28 (8.7%) patients. Cardiovascular and renal complications were also common (see Table 3).

**TABLE 3:** Documented number of complications in reviewed files (*N* = 323).

Complication	Frequency	Percentage
Foot complications	28	8.7
Cardiovascular	24	7.4
Nephropathy	22	6.8
Retinopathy	19	5.9
Erectile dysfunction	2	3.7
Others	3	0.9
Unknown	225	69.7

In the performance of routine investigations and screening for target organ damage, at each clinic visit, 99% of all patients had blood glucose tested and documented at every clinic visit. A total of 164 (50.65%) patients had HbA1c tested the previous year, and of these only 14 (4.3%) had levels < 7%. Six-monthly HbA1c was done for 40 (12.4%) patients, and only three patients had their HbA1c assessed more than twice that same year (Table 4).

**TABLE 4:** Documented routine investigations, glycaemic and target organ damage in reviewed files (*N* = 323).

Examinations	Done (*n*)	Percentage
HGT	322	99.7
3 monthly HbA1c	3	0.9
6 monthly HbA1c	40	12.4
Annual HbA1c	164	50.8
Annual serum creatinine	179	55.4
Annual lipogram	154	47.7
Urine dipsticks	164	50.8
Urine Albumin: creatinine ratio	11	3.4

HbA1c, glycated haemoglobin test; HGT, hemo glucose test.

**TABLE 5:** Adequacy of diabetes control according to Society for Endocrinology Metabolism and Diabetes South Africa 2017.

Indicator	SEMDSA cut off	Number above cut off	Proportion (%)	Mean	s.d.	Median	Range
HbA1c	≥ 7	111/164	67.6	9.97	2.80	9.60	12.7
Serum creatinine (µmol/L)	≥ 90	87/179	48.6	81.20	38.06	71.00	304
Total cholesterol (mmol/L)	≥ 4.45	55/154	35.7	4.45	1.22	4.30	5.68
LDL (mmol/L)	> 1.8	96/154	62.3	2.28	0.96	2.35	4.71
HDL (mmol/L)	≤ 1.2	100/154	64.7	1.28	0.41	1.30	1.91

SEMDSA, Society for Endocrinology Metabolism and Diabetes South Africa; s.d., standard deviation; HbA1c, glycated haemoglobin test; LDL, low-density lipoprotein; HDL, high-density lipoprotein.

A total of 154 (47%) patients had an annual lipogram test. Ninety nine of these 154 (64%) had total cholesterol levels less than 4.5 mmol/L. Low-density lipoprotein (LDL) test results were consistently greater than 1.8 mmol/L in 62.3% in these patients. More than half of patients had serum creatinine (µmol/L) and eGFR measured and documented. Of these, only 51% had normal results. A total of 164 (50.6%) patients had urine dipsticks done, but only 11 of these patients had urine albumin-creatinine ratios (urine ACrs) assessed.

## Discussion

In this study, the compliance of doctors with diabetic treatment guidelines in the management of diabetic patients in a district hospital is explored. The results show poor compliance across various aspects of the SEMDSA guidelines. Markers of control and other process-of-care indicators were either not being done or not being documented.

According to the International Diabetes Federation,^1^ DM is on the increase in developing countries. The consequences of poor compliance with diabetic treatment guidelines are increases in morbidity and mortality, resulting in poorer outcomes.

Glycaemic control as measured by HbA1c is the single most important factor in assessing the control in diabetic patients because it has a strong predictive value for diabetic complications and is thus the best indicator of the effectiveness of diabetes care.^26^ Most of the research on diabetes has shown that diabetic complications are directly related to glycaemic levels and the prevalence of diabetic complications sharply and significantly increase as the glycaemic levels rise.^27^ The recommended glycaemic target is 7%, and every 1% above this level has been associated with a 38% – 40% higher risk of micro and macrovascular complications, as well as death.^27,28^ The findings of this study in terms of glycaemic monitoring and control also show that irregular monitoring is likely to contribute to poor glycaemic control.

These negative findings are similar to various local studies in South Africa and many countries in the African continent.^14,21,22^ Research suggested that less developed countries, for example, Mexico, also have poor compliance to guidelines.^29^ In contrast, in developed countries, glycaemic monitoring in United States and Europe was done at recommended intervals showing a higher compliance rate with guidelines.^30,31^ Developed countries have a higher doctor to patient ratio^32^ that could contribute to more time per consultation and probably better compliance and/or documentation of the consult. The researcher acknowledges the differences in healthcare systems and resources in these countries but also hypothesised that a more structured approach to diabetic care and a higher doctor-to-patient ratio compared with their South African counterparts could have contributed to better compliance and management of DM in developed countries.

The consequence of poor glycaemic monitoring and control are complications. Diabetic foot problems were the most common complication found in this study, and it is interesting to note that all patients who had a documented foot exam had abnormal findings. The SEMDSA guidelines were designed to prevent and limit complications when regular screening is done. However, in this present study, it is unclear if the foot exams were done due to complaints or following guidelines. Even though the present study did not establish the duration of DM, the risk of diabetic peripheral neuropathy (DPN) increases with the duration of diabetes, and its presence is associated with the presence of microalbuminuria and diabetic kidney dysfunction.^33^ Further, one in every five individuals living with diabetes is likely to have diabetic neuropathy, with the risk of severe neuropathy evolving to amputations in about 6% of the diabetic population.^33,34^ In addition, peripheral neuropathy causing DM foot problems has been associated with sexual dysfunction in men due to the microvascular damage in DPN also causing damage to the penile vasculature. Thus, the lack of screening in diabetic peripheral neuropathy suggests that other signs of severe microvascular damage, like erectile dysfunction (ED), are also being missed.^35^ It is thus imperative that medical practitioners strengthen and improve the evaluation and diagnosis of DPN, because timely screening by regular foot examinations will enable earlier detection of foot problems and the instituting of timeous interventions.

Interestingly, even though more than 50% were male patients, only two patients had ED documented. However, sexual dysfunction cuts across gender lines and is a commonly missed symptom in patients with chronic conditions.^36^ Further, ED is common among people living with DM, and an additional biomarker for coronary events.^37^ The prevalence of ED is estimated to be as high as 70% in Africa, and the risk increases with just 1 year of being diabetic and is made worse in the setting of poor glycaemic controls.^38^ During consultations with patients, sexual history is often not taken by healthcare workers; thus patients living with sexual dysfunction are missed. In a study done to assess care among patients living with chronic disease in the North west province of South Africa, the examination of sexual dysfunction was found to be grossly neglected. Nearly all the male patients had sexual dysfunction and more than 80% of the female patients had symptoms suggestive of sexual dysfunction.^39^ In other studies done by Pretorius et al., sexual symptoms were investigated by way of enquiry in only two patients of the total number consulted.^40,41^ This, therefore, indicates that a significant number of patients with ED may have been missed or alternatively not being documented, and, therefore, they are most likely not receiving the care they deserve. This healthcare check warrants the appropriate regular screening and intervention for sexual dysfunction in all patients living with DM regardless of sex. Developing effective communication skills and adequate training of healthcare workers to enable them to identify patients with sexual dysfunction would be a positive step in eliciting this complication and institute targeted treatment for people living with DM.

Significantly, the increasing prevalence of DM has led to an increase in diabetic kidney disease. Microalbuminuria is often used as an early marker for diabetic kidney disease detected via the urine ACr and proteinuria (urine dipsticks) as the hallmark of kidney disease.^42^ Besides glycaemic monitoring, renal function monitoring has been identified as the main factor in the preventing the development of renal failure.^43^ Furthermore, in the presence of diabetes, there is a more rapid decline in renal function worsening with poor control. This complication could be minimised in the first place if the urine albumin-creatine ratio test is combined with other aspects of the kidney function test, as it is a useful way to identify early kidney dysfunction, institute treatment and slow down further decline. However, given the low inclination to screen for microalbuminuria among patients in this present study, combined with the setting of poor glycaemic controls, it is very likely that many patients with progressive diabetic renal failure are being missed who could have benefited from early referral to nephrologists to slow progression to end stage renal disease.

**FIGURE 1:**
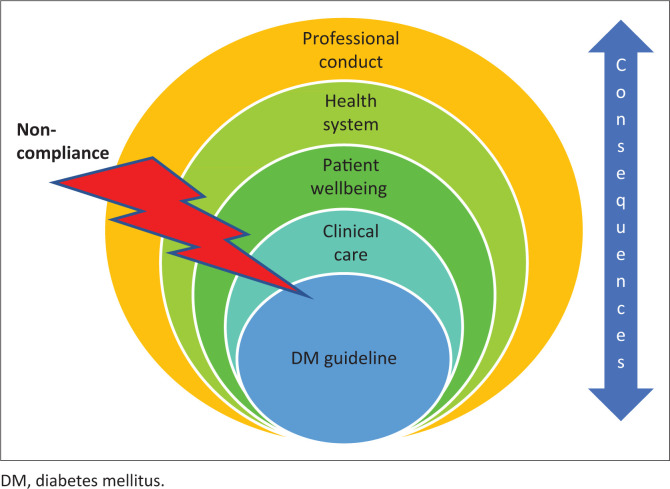
Multi-layered consequence of non-adherence with diabetes mellitus guidelines.

The findings of this study are limited to compliance with a guideline; however, the consequence of non-compliance is multi-layered. The findings of this study are to be seen in the direct and indirect implications of non-compliance with guidelines, as shown in Figure 1.

The findings of this study have a huge impact on not only clinical care as discussed but also on patients’ wellbeing, the health system and the healthcare providers’ professionalism. For the patient, there is an increase in missed workdays leading to job losses, an increase in the number of years lost due to resulting disability and overall, a reduction in quality of life.^44^ Not only is a patient failed, but an entire family and eventually a community. The effects are also not lost on the healthcare worker who is open to litigation on account of substandard care.^23,24,45^ It may be that care was given but if not documented could be interpreted legally as not done.^46^ In addition, the effect on the healthcare system is enormous. The financial cost needed to care for DM patients with complications from DM, such as prosthesis for foot amputees, dialysis for renal complications to name a few exerts further burden on the already strained healthcare system.^47^ Adequate care given to patients by complying with guidelines could help minimise these negative consequences.

This study highlighted the non-compliance of health workers with diabetic treatment guidelines in a West Rand district hospital. Physician-related factors and healthcare system were identified to contribute to poor compliance with treatment guidelines by medical practitioners as well as the guideline implementation process.^48^ Upon reflection, the researcher could relate some of these factors identified in other research, also could apply to this study, such as inadequate numbers of staff, professional failure to keep up with latest guidelines, excessive patient load, overburdened healthcare workers resulting in burnout, poor organisation and patient flow. Further research is needed to study some of the mentioned factors to establish what is needed to improve the overall care of diabetic patients. In the researcher’s opinion, the above-mentioned factors can be mitigated by the medical practitioners updating themselves with latest clinical guidelines, implementing a booking system in the healthcare system to prevent clogging of patients on certain days, ensuring that guidelines are widely disseminated and readily available to medical practitioners.

### Limitations

Convenience samples have low external validity or generalisability; therefore, the study must be interpreted within the context of the study. Using files in a consecutive manner limited sampling, selection and researcher bias that are common in convenience sampling. Failure to document examination or investigation findings were reported as not done, even if the patient might have had the benefit of the service, as this study reflects on recorded data.

## Conclusion and recommendation

Compliance with DM guidelines was poor in this study despite evidence that guidelines ensure good standard of care, decrease the risk for complications and generally provide better health outcomes for patient. Not following guidelines put the patient and the profession at risk.

In addition, it is imperative that there is a review of how treatment guidelines are implemented, disseminated and utilised to improve patient management and outcomes. Therefore, recommendations from this study include more studies required to assess possible barriers to compliance with guidelines by medical practitioners and encourage regular quality improvement cycles to improve medical-practitioner actions.
